# Impact of different enzymes on biofilm formation and mussel settlement

**DOI:** 10.1038/s41598-022-08530-4

**Published:** 2022-03-18

**Authors:** Jiazheng Li, Chi Zhang, Xiaomeng Hu, Asami Yoshida, Kiyoshi Osatomi, Xingpan Guo, Jin-Long Yang, Xiao Liang

**Affiliations:** 1 International Research Center for Marine Biosciences, Ministry of Science and Technology, Shanghai Ocean University, Shanghai, China; 2 Shanghai Collaborative Innovation Center for Cultivating Elite Breeds and Green-Culture of Aquaculture Animals, Shanghai, China; 3 Southern Marine Science and Engineering Guangdong Laboratory, Guangzhou, China; 4 Graduate School of Fisheries and Environmental Sciences, Nagasaki University, Nagasaki, Japan

**Keywords:** Ecology, Ecology

## Abstract

Enzymes have been known to impact the biofilm forming capacity. However, how the enzymes mediate the biofilm formation and macrofouling remains little known. Here, we investigated the effects of the three kinds of proteases, four kinds of glycosidases and one kind of lipase on the detachment of biofilms of *Shewanella marisflavi* ECSMB14101, identified biofilm total proteins response to enzyme treatments, and then tested the effects of biofilms treated with enzymes on the settlement of the mussel *Mytilus coruscus* plantigrades. The results showed that the cell density of bacteria in biofilms formed at different initial bacterial density were noticeably reduced after treating with all tested enzymes, and Neutrase and α-Amylase exhibited best removing efficiency of > 90%. Bacterial total proteins in *S. marisflavi* biofilm noticeably reduced or disappeared after treated by Alcalase. For the settlements of the mussel *M. coruscus* plantigrades, inducing capacities of *S. marisflavi* biofilm were noticeably suppressed and downregulation was > 75% at the initial density of 5 × 10^6^ cells/cm^2^. Thus, the tested enzymes could effectively remove the adhered bacterial cell, inhibit the biofilm formation and finally suppress the mussel settlement. Our findings extend novel knowledge to developing eco-friendly approach to control micro- and macro-fouling.

## Introduction

Biofilms are mainly composed of surface-associated microbial communities and their secreted extracellular polymers, as well as some organic or inorganic particles^1–3^. As the initial microorganism colonies on the surface, bacteria have been proved that they could induce recruitment of many macrofouling invertebrates including mussels^4–7^. Previous studies have found the biofilm of *Shewanella* sp. has an obviously inducing activity for marine invertebrates^8,9^, thereby both of them can cause the harm of microfouling and macrofouling^10^.

To reduce or eliminate the harm of fouling organisms, one useful way is to inhibit or reduce the initial settlement organisms and then affect the settlement of macrofouling organisms subsequently^11^. The techniques of surface antifouling mainly using paints containing copper, tin, insecticide, oxide or chloride compounds, and some new antifouling coatings were also used currently, such as paints containing nano materials^12,13^. However, most of them could exist in the marine environment for a long time and toxicity to many marine organisms^14^. Therefore, it is essential to search environment-friendly antifouling materials. Hydrolases have many characters, such as the mass production easily, the biological degradation efficiently, the species diversity and the weak toxicity as well as they could degrade the matrix molecules. However, whether these enzymes could be used in degrading extracellular polymeric substances (EPS) on biofilms or cell wells and destroying the structure of the compounds, such as the Quorum sensing signal molecules, remains little known. This may be able to control or reducing the biofouling.

The mussel *Mytilus coruscus* is a typical macrofouling and commercial species in China^15–18^. It could settle on the ship, the water pipes, cooling facilities, port construction or other marine facilities to cause some fouling damages. So, it can be used as the modes of fouling organisms^7,9,15–18^. Therefore, the present study will investigate the effect of the three kinds of proteases, four kinds of glycosidases and one kind of lipase on the detachment of biofilms of *Shewanella marisflavi* ECSMB14101^9,19^ and then test the effects of biofilms after enzymes treated on the mussel settlement. Meanwhile, the change of biofilm proteins before and after enzymes treatment was also examined.

## Results

### Enzyme activities

**Figure 1:**
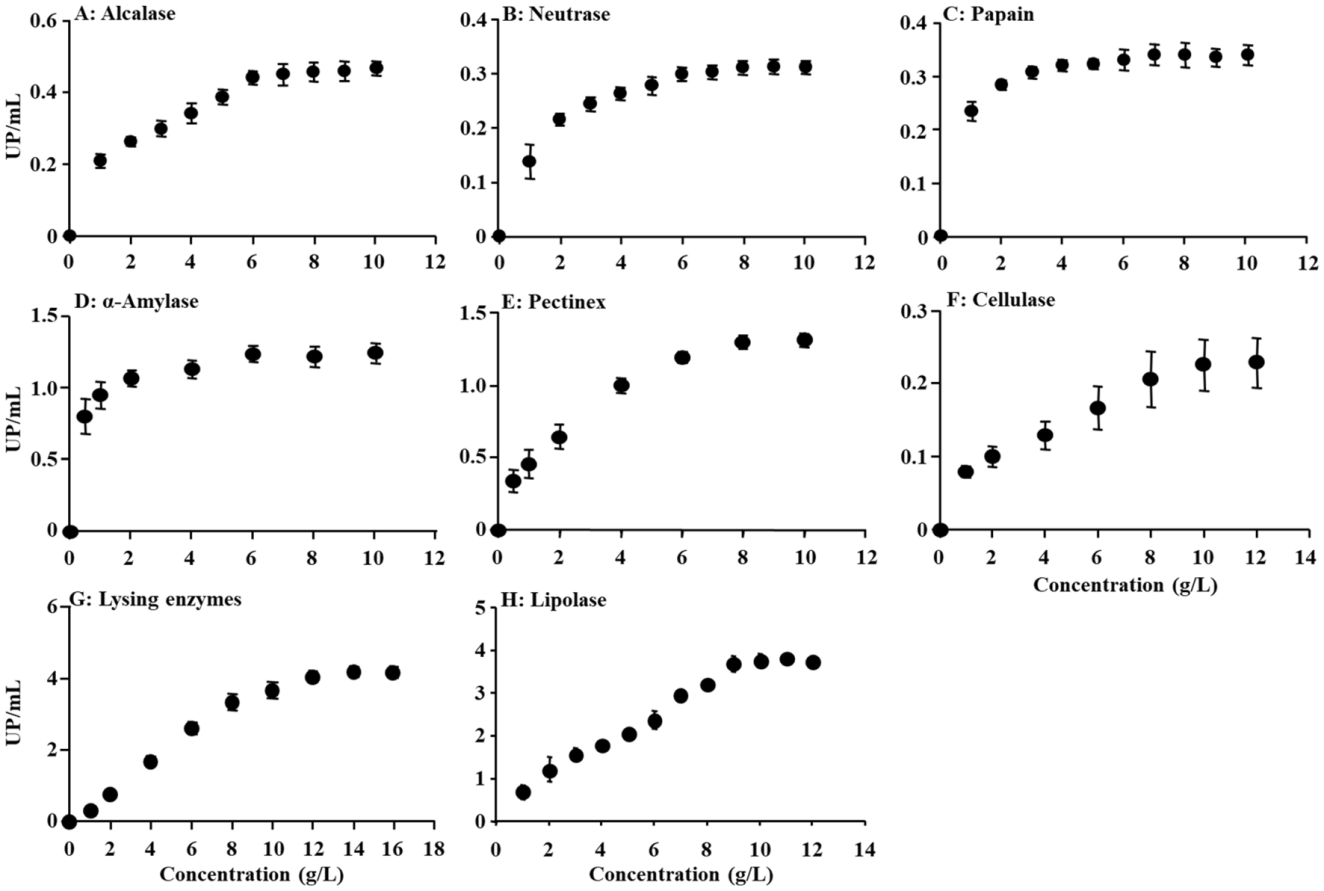
The activity of proteases, glycosidases and lipase in different concentrations.

The activities of three kinds of protease were shown in Fig. 1A–C. The activity of Alcalase showed a rising trend in the concentrations ranging from 0–6 g/L, and the higher activity about 0.44 at the concentration of 6 g/L in which the reaction was reached equilibrium (Fig. 1A). No variance of the activity was observed among various concentrations (*P* > 0.05, Fig. 1A). The activities of Neutrase and Papain had similar tendency of Alcalase and the suitable concentration of the two enzymes were 8 g/L and 7 g/L, respectively (Fig. 1B and C). Similarly, no variance of the two enzyme activities was observed among various concentrations (*P* > 0.05; Fig. 1B and C).

The activities of four glycisides were shown in Fig. 1D–G. There was no significant difference between the activities of the four enzymes at the different concentrations (*P* > 0.05). The activity of α-Amylase was reached equilibrium state at the concentration of 6 g/L. Meanwhile, the activities of Pectinex and Cellulase were increased gradually with the increasing of enzymes concentration, but their period of stable were not obviously (Fig. 1E, F). So, the activities of them were acquired at the concentration of 10 g/L and 8 g/L, respectively. The activities of Lysing enzyme obviously increased, and then showed stable change with the increasing concentration of enzymes (Fig. 1G). The reaction was kept in balance at 12 g/L with enzyme activity of 4.07. The activity of Lipolase was shown in Fig. 1H. No variance of the activity was observed among various concentrations (*P* > 0.05). Meanwhile, the activity of lipolase was changed slowly at the concentration of 10 g/L and the activity was about 3.78 at this time.

### Impacts of enzymes on *S. marisflavi* biofilm formation

**Figure 2:**
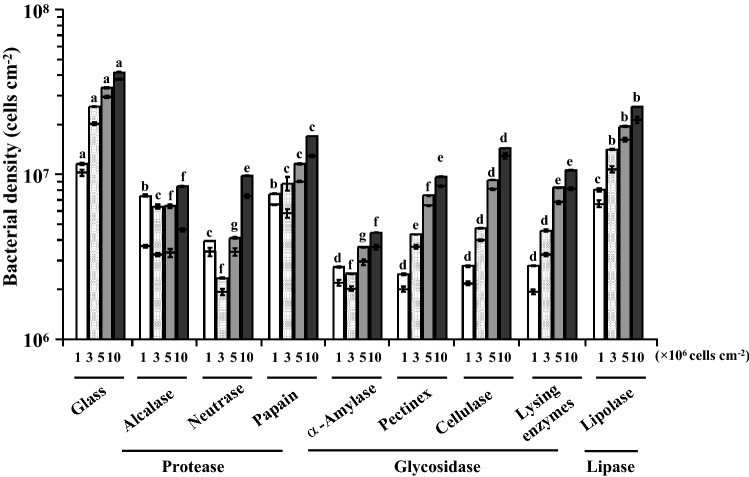
Bacterial density in *S. marisflavi* biofilm with different enzyme treatments.

**Figure 3:**
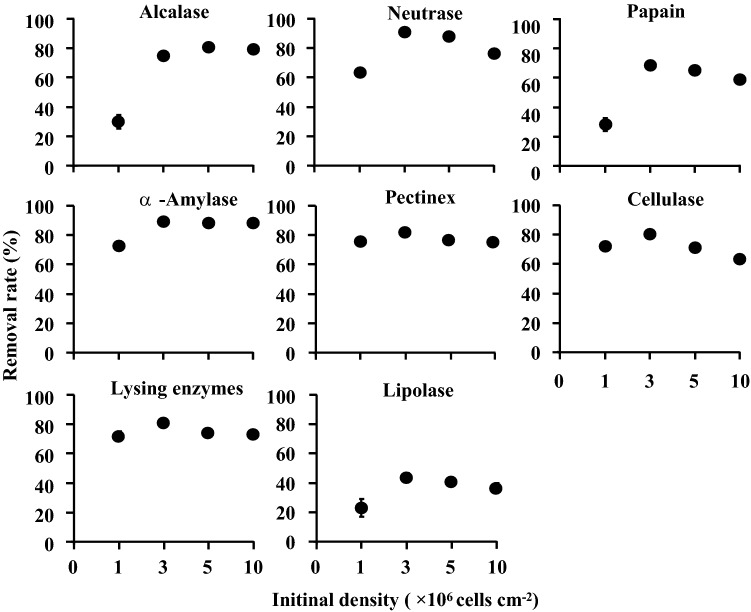
Removal rate on *S. marisflavi* biofilm with different enzyme treatments.

**Figure 4:**
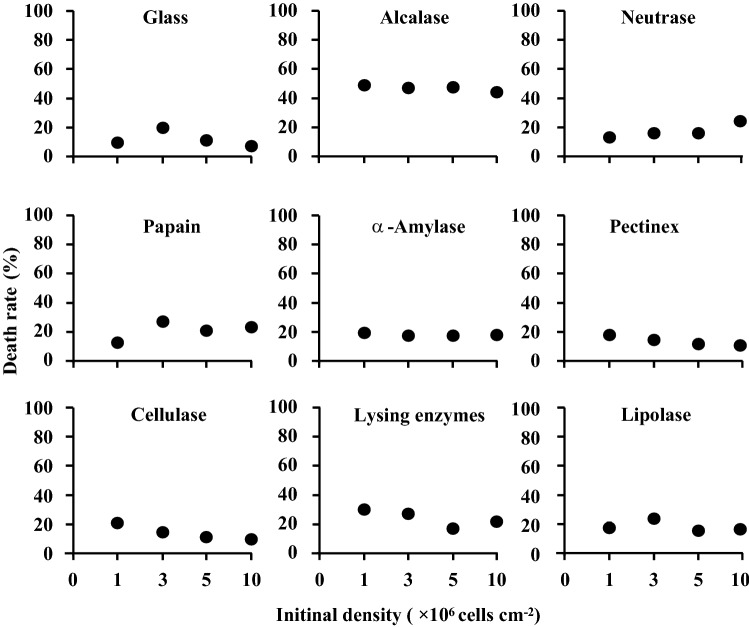
Dead bacterial density on *S. marisflavi* biofilm with different enzymes treatments.

Cell densities of treated and untreated *S. marisflavi* biofilms were shown in Fig. 2. The initial bacteria density of the four groups were 1 × 10^6^, 3 × 10^6^, 5 × 10^6^ and 10 × 10^6^ cells/cm^2^, respectively. As the results shown, in the initial concentration of 1 × 10^6^ cells/cm^2^, the density of bacteria in the biofilm treated with enzymes changed significantly (*P* < 0.05). The treatment with Pectinex was the most obvious result with bacterial density of 3 × 10^6^cells/cm^2^ and the removal rate of 78.34% (Fig. 3). However, there was no significant difference on the bacterial densities in the biofilms treated with four glycosidases (*P* > 0.05). The death rate was highest in the group treated with Alcalase and the rate was 50.14% (Fig. 4).

In the initial concentration of 3 × 10^6^ cells/cm^2^, the bacterial densities in *S. marisflavi* biofilms treated and untreated with enzymes had significant differences between each other (*P* < 0.05, Fig. 2). The treatment effects were best by Neutrase and α-Amylase with minimum bacterial density. The corresponding removal rates were 90.9% and 90.28%, respectively (Fig. 3). In addition, the death rates were relatively high in the groups treated with Lipolase, Lysing enzymes and Alcalase. The highest rate reached 48.8% by Alcalase (Fig. 4). Significant differences of the bacterial densities between the treated and untreated groups at the initial concentration of 5 × 10^6^ and 10 × 10^6^cells/cm^2^ were found (*P* < 0.05; Fig. 2). The density was lowest in the group treated by Neutrase and α-Amylase, respectively (Fig. 2). Compared with another seven kinds of enzymes, the Alcalase had the best treatment effect and the death rate reached 45.22% and 43.36%, respectively (Fig. 4).

### Images and total proteins of bacteria on the biofilms treated by Alcalase

**Figure 5:**
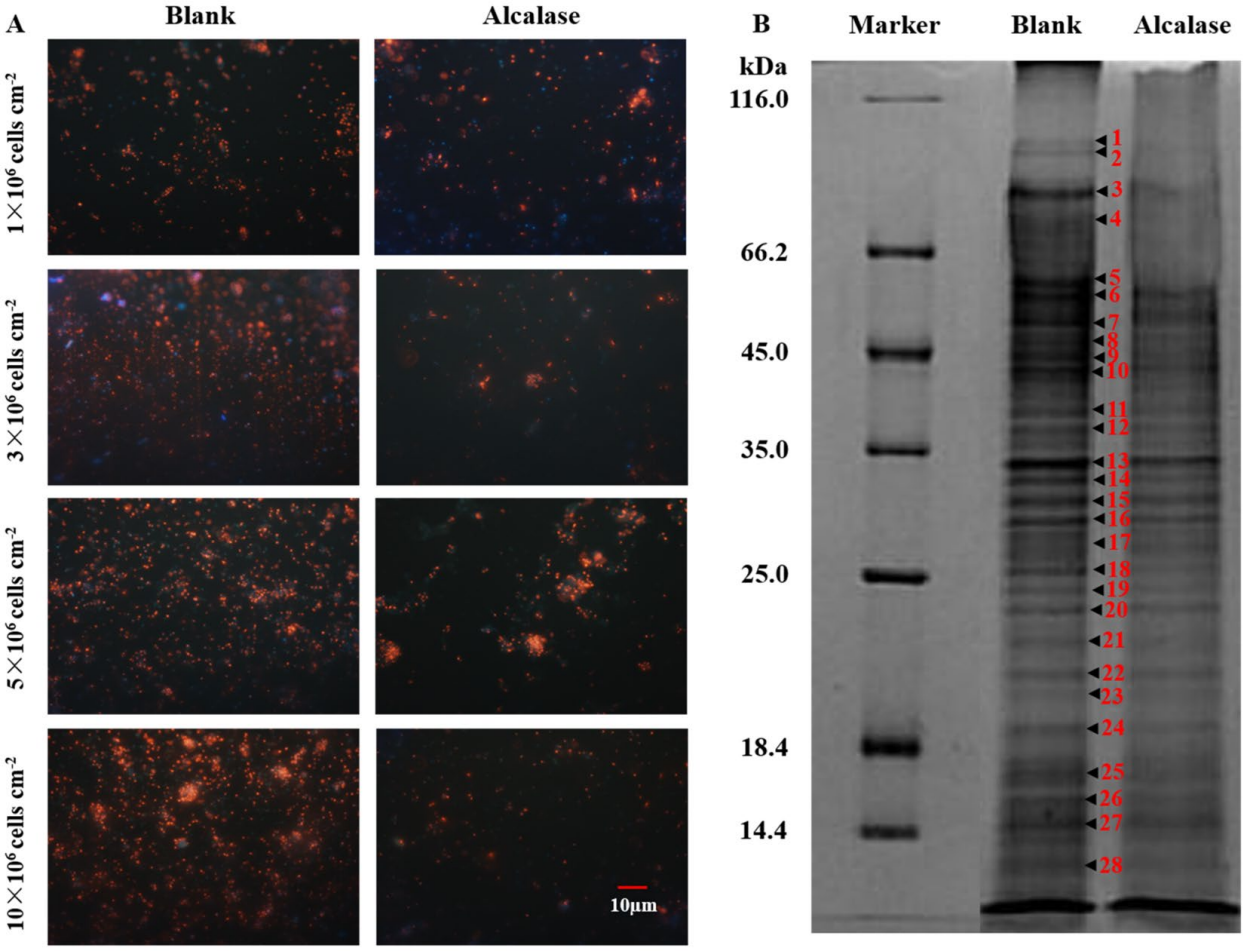
The images of *S. marisflavi* biofilms (**A**) under BX51 and biofilm bacterial proteins by SDS-page (**B**) treated by Alacalse.

The images of *S. marisflavi* biofilm were shown in Fig. 5A. As the results shown, in all groups with initial bacterial density of 1 × 10^6^, 3 × 10^6^, 5 × 10^6^ and 10 × 10^6^ cells/cm^2^, after treating with Alcalase, the bacterial density was reduced obviously and the ratio of living and dead bacteria was also changed significantly.

The analysis of the total bacterial proteins extracted from the biofilms treated by Alacalse were shown in Fig. 5B and Fig. S1. The image of the SDS-page shown that the changes of the total proteins were obviously and some protein bands reduced or even disappeared. The gray values and the molecular weights were calculated by the software of CS Analyzer 3.0 (ATTO, Japan) (Table 1). Analysis of the gray values indicated that three bands (No. 1, 4, 18) were almost disappeared with the gray value decreased more than 80% in the detected 28 bands. The gray values decreased less than 20% were deemed as no change and the bands number of 7, 22, 23, 27, 28 were liked this. Other bands were decreased in different degree.

**Table 1:** The SDS-page analysis of biofilm bacterial proteins after Alcalase treatment.

No.	Experimental	Contents		
	MW (kDa)	Control	Treated	T/C (%)
1	98,136	4515	770	17
2	95,275	5005	1330	27
3	81,541	22,855	9835	43
4	71,063	36,120	3430	9
5	58,287	15,050	6160	41
6	55,914	22,295	11,025	49
7	51,184	20,055	24,255	121
8	46,911	14,945	5740	38
9	44,518	16,975	9415	55
10	43,103	20,125	13,090	65
11	38,667	5845	3850	66
12	36,904	7000	2835	41
13	33,886	32,725	15,995	49
14	32,386	15,785	4130	26
15	30,667	18,725	10,430	56
16	29,093	15,365	7245	47
17	27,500	21,455	7770	36
18	25,443	9205	1365	15
19	24,436	5635	1260	22
20	23,600	7665	5285	69
21	22,279	8855	4935	56
22	20,991	7000	6965	100
23	20,221	3360	3325	99
24	18,980	9590	4130	43
25	16,991	23,520	10,290	44
26	15,674	11,865	5915	50
27	14,727	24,955	20,440	82
28	12,711	7315	7105	97

### Mussel settlement on *S. marisflavi* biofilms treated with enzymes

**Figure 6:**
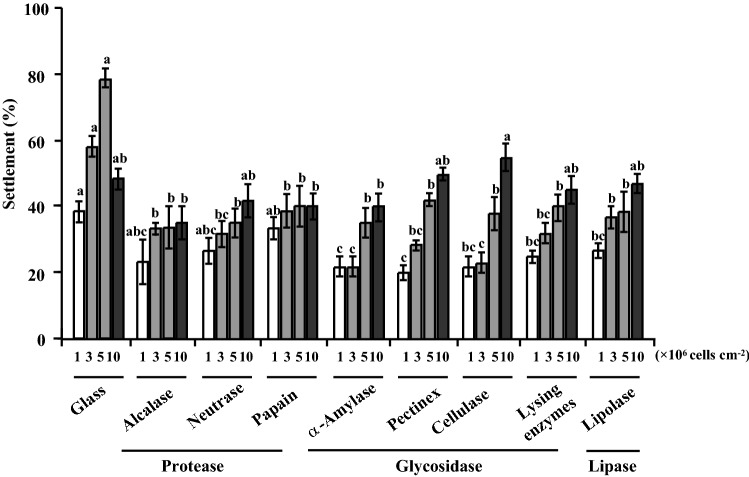
Percentage of mussel settlement on *S. marisflavi* biofilms treated with different enzymes. Small letter indicates significant variance (*P* < 0.05).

The plantigrade settlement rate was only 18% in Glass without biofilms. In Fig. 6, in the initial concentration of 1 × 10^6^ cells/cm^2^, the settlement of plantigrades in positive control group (untreated biofilms) was highest, with a rate of 38%. There was no variance between the groups of positive control and three protease treatments (*P* > 0.05), but the settlement of plantigrades in other groups with five enzymes had significant differences with the positive control (*P* < 0.05). What’s more, the activities of *S. marisflavi* biofilms treated with α-Amylase and Pectinex decreased obviously (*P* < 0.05) and the settlement were 22% and 20%, respectively.

In the initial concentration of 3 × 10^6^ cells/cm^2^, the settlement of plantigrades in all enzyme-treated groups were noticeably reduced in comparison to untreated groups (*P* < 0.05), and the group treated with α-Amylase had the best inhibitory activity with a rate of 22%. When the initial concentration was 5 × 10^6^ cells/cm^2^, the untreated biofilm had the highest inducing activity of 78%, which was noticeably greater than enzyme treated biofilms (*P* < 0.05). In the initial concentration of 10 × 10^6^ cells/cm^2^, there was no variance among the biofilm groups treated with Neutrase, Pectinex, Lysing enzymes, Lipolase and untreated groups (*P* > 0.05). Moreover, the group treated with Cellulase had a high inducing activity of 55%.

## Discussion

In the present study, the eight enzymes can reduce bacteria in the *S. marisflavi* biofilms formed from the different initial bacterial densities. The effects of Alcalase, Neutrase and α-Amylase were better than other enzymes on reducing bacteria. Meanwhile, the residual bacteria density and the ratio of living bacteria and death bacteria in the biofilms treated with Alcalase was lower than those treated with Neutrase and α-Amylase. These results indicated that Alcalase had an obviously effect on the death of bacteria in biofilms.

The biofilms are mainly composing of bacteria and their secreting EPS^20,21^. There are two ways for enzymes acting on bacterial biofilms, one of them is to get into cells to make bacteria death directly, other way is the enzymes acting on the EPS, and all of them are aim to degrade the biofilms^22^. As the results shown, the mechanism of Alcalase maybe relate to the enzymes directly acting on the cells of bacteria which had a high death rate. Meanwhile, the results of SDS-page also proved that the enzymes could degrade some proteins in the cells which were similar to Pettitt’s research^23^. The lower bacterial density and death ratio of biofilms treated by other seven kinds of enzymes maybe caused by the second mechanism as well as by combing with the two kinds of mechanisms. Further researches should be conducted by analyzing the change of EPS.

In many previous studies, enzymes have inhibitory effects on the formation of biofilms^24^, such as pathogenic bacteria^25,26^, oral microbial biofilms^27,28^ and industry biofilms^14^. However, the potential of these enzymes in the marine environment is still relatively less known. The results shown that the hydrolases could inhibit the formation of *S. marisflavi* in marine environment and this may be due to the proteins on the biofilms, but what those proteins in present study were still unknown. Fortunately, the commonly used methods of two-dimensional electrophoresis and mass spectrometry will be applied to analysis the proteins.

Enzymes had obviously inhibition effects on the settlement of some invertebrates^22,23,29^, such as barnacles. That maybe due to the QS was hydrolyzed by enzymes or produced some chemicals that could suppress recruitment of benthic animals^29^. The present study had demonstrated that enzymes could not only affect the formation of bacterial biofilms but also further inhibit the mussel recruitment. Based on the mechanism of release coating^12,30–34^, the enzymes with inhibitory effect, could be mixed in various coatings and then applied for marine antifouling. But the complicated marine environment with the change of temperature^35^, salinity or pH^36^ may affect the activity of enzymes or cause the inactivation of some enzymes^37^. The retention time of the enzymes in the coating determined by the above conditions. In addition, the enzymes could also affect other creatures in short time in the marine environment which may affect the balance of the whole ecosystem^38^. Thus, it is very essential to develop more eco-effective materials, such as enzymes.

In conclusion, the results confirmed that the effect of detaching biofilms of *S. marisflavi* were related to the kinds of enzymes and the density of bacteria on biofilm. Simultaneously, the change of bacterial biofilm may lead to the variance in the settlement of plantigrades. Therefore, the study of enzymes was important in the fields of microfouling and macrofouling.

## Materials and methods

### Enzymes and enzymatic activities

In the present study, the enzymes were bought from Sigma or Novozymes, three kinds of proteases, four kinds of glycosidases and one kind of lipase were examined and some characteristics of those enzymes were shown in Table 2.

**Table 2:** Tested enzymes and their respective characteristics.

Enzyme	Manufacturer	Microorganism source	EC number	pH	Forms
Alcalase	Novozymes	*Bacillus Licheniformis*	EC3.4.21.62		Liquid
Neutrase	Novozymes	*Bacillus amyloliquefaciens*	EC3.4.24.4	7	Liquid
Papain	Sigma	*Papaya latex*	EC3.4.22.2	6–7	Powder
α-Amylase	Sigma	*Bacillus subtilis*	EC3.2.1.1		Powder
Pectinex	Novozymes	*Aspergillus aculeatus*	EC3.2.1.15	3.5	Liquid
Cellulase	Sigma	*Trichoderma reesei*	EC3.2.1.4	4.5–6	Liquid
Lysing enzyemes	Sigma	*Trichodermaharzianum*	EC3.2.1.58		Powder
Lipolase	Sigma	*Thermomyces lanuginosus*	EC3.1.1.3		Liquid

All the experiments for determining the enzyme activity were conducted in autoclaved filtered sea water (AFSW) with pH = 8.15 at 18 °C. The concentration of each enzyme with the maximum activity was used to treat the *S. marisflavi* biofilms. Protease activity was measured by the method with a minor modification^39^. In details, as substrate, the 2% (w/v) of azocasein was placed in AFSW at 18 °C, and then protease was applied to this substrate at concentrations (0.5–10 g/L) for different incubation times (3–12 min). The reaction ended through addition of 10% trichloroacetic acid (TCA), and then took a suitable amount of supernatant liquid after centrifuging for 30 min at 3500 rpm to react with Folin-Ciocalteu’s phenol Reagent for 20 min under the condition of water bath at 18 °C. The casein concentration was determined spectrophotometrically (UNIC 2100spectrophotometer) at 450 nm. The blank control group changed the order of adding protease and TCA solution. Protease activities were evaluated as the amount of enzyme required to generated one μmol casein per minute.

Glycosidase activity was determined via the amount of released sugars during the hydrolysis of the appropriate substrate by enzymes^11,40,41^. The reaction was started with the mixture enzyme liquid at 0–18 g/L in phosphate-citrate buffer (pH: 8.15), and was then added 12.5 g/L substrate in AFSW at 18 °C for incubation. The soluble starch, pectin, carboxymethy cellulose and β-glucan were as substrates of α-Amylase, Pectinase, Cellulose and Glucanase respectively. The blank control group changed the order of adding substrate and DNS solution. Enzymatic activity was determined via enzyme content required to produce 1 μmol sugar min^−1^.

Lipase activity was determined by using Lipase Activity Assay Kit. The concentrate of Lipase was diluted with AFSW to tested concentrations (1–12 g/L), and then acted on the substrate with the concentration of 465.35 μmol/L in AFSW at 18 °C (pH: 8.15) for incubation. Finally, the absorbance was detected at 420 nm. Enzymatic activity was defined as the amount of enzyme required to produce one μmol sugar per minute.

### Mussel culture

*M. coruscus* plantigrades were collected from Shengsi Island in the East China Sea, Zhoushan, Zhejiang province. The length and height of the palntigrades were 780.3 ± 0.5 µm and 440.5 ± 0.5 µm, respectively. Plantigrades of *M. coruscus* were fed with *Chaetoceros gracilis* in darkness at an incubator maintained at 18 °C.

### The bacteria for the formation of biofilm

Stock cultures of *S. marisflavi* ECSMB14101^9,19^, were obtained from the storage bacteria from our laboratory. This species was isolated and purified by the ZoBell 2216E agar plate, which came from the natural biofilms in the sea, Zhoushan Zhejiang province. Previous studies have found that this species has an obviously inducing activity of *M. coruscus* recruitment^9,42,43^.

### Formation of *S. marisflavi* biofilms and treated with enzymes

The formation of monospecific bacterial biofilm according to our methods^9,17,44^. *S. marisflavi* was cultured in ZoBell broth. The bacterium *S. marisflavi* were resuspended in AFSW to form the bacteria concentrated solution. The bacteria solution and AFSW into sterile glass Petri dishes with a half of a sterile glass microscope slide (38 mm × 26 mm) to form the bacteria biofilm with varying initial bacterial density at 18 °C for two days. Six replicates of each density were conducted.

The *S. marisflavi* biofilm on the glass slide was washed to clean unattached cells up and then immersed into Petri dishes with enzymatic preparation (20 mL) at the desired concentration for 1 h. Then, they were rinsed three times with AFSW gently for the later experiments.

### Mussel settlement bioassays

Plantigrades of 10 individuals were kept on glass Petri dishes containing an untreated or treated *S. marisflavi* biofilm as well as 20 mL of AFSW. The blank controls used the sterile glass slides. The settlement inducing activity of biofilm was determined by settlement rate of plantigrades on *S. marisflavi* biofilms after 6 h, 12 h, 24 h, 48 h.

### The detachment of adhered bacteria and determination of bacterial cell survival and density

The detachment of adhered bacteria by enzyme treatments was according to a previous method^11^ and bacterial survival and density were described by Yang et al.^9^. The treated and untreated *S. marisflavi* biofilms were all rinsed three times as before and then stained. Amounts of viable and total bacteria on *S. marisflavi* biofilms were counted. Three biological replicates were set up.

### Analysis of the total bacterial proteins

The treated and untreated bacterial biofilms were collected, centrifuged and washed twice with 1% PBS. After *S. marisflavi* cells were disrupted by sonication for 20 min at 4 °C, the mixtures were rotated by WH-986 Rotation Mixer for 2 h at 4 °C and then the total proteins in the supernatant were collected by removing the precipitation after centrifugation. The proteins were quantified using the RCDC method. The resulting protein patterns were captured and evaluated through Bio-rad Image lab software (Hercules, CA, USA).

### Data analysis

The percentage of plantigrade settlement rate was arcsine-transformed and tested for normality using JMP software. Differences were considered significant at *P* < 0.05.

## Supplementary Information

Supplementary Figure S1.
