# Supplementary material for: Impact of different enzymes on biofilm formation and mussel settlement
Source: Sci Rep. 2022 Mar 18;12:4685. doi: 10.1038/s41598-022-08530-4 (PMC8933495; doi:10.1038/s41598-022-08530-4)
Supplement: Supplementary file 1 — Supplementary Figure S1. [file 41598_2022_8530_MOESM1_ESM.pdf]

# Impact of Different Enzymes on Biofilm Formation and Mussel Settlement

Jiazheng Li[1, 2, 3*], Chi Zhang[1, 2, 3*], Xiaomeng Hu[1, 2, 3], Asami Yoshida [4], Kiyoshi Osatomi [4], Xingpan Guo[1, 2†], Jin-Long Yang [1, 2, 3], Xiao Liang[1, 2, 3†]

[1] *International Research Center for Marine Biosciences, Ministry of Science and Technology, Shanghai Ocean University, Shanghai, China*

[2] *Shanghai Collaborative Innovation Center for Cultivating Elite Breeds and Green-culture of Aquaculture animals, Shanghai, China*

[3] *Southern Marine Science and Engineering Guangdong Laboratory, Guangzhou, China*

[4] *Graduate School of Fisheries and Environmental Sciences, Nagasaki University, Nagasaki, Japan*

Running head: Mussel settlement on enzyme-treated biofilms

[†] Corresponding author. E-mail: guoxingpan2008@163.com, x-liang@shou.edu.cn

Tel: + 86-21-61900286; Fax: + 86-21-61900280

[*] These authors contributed equally.

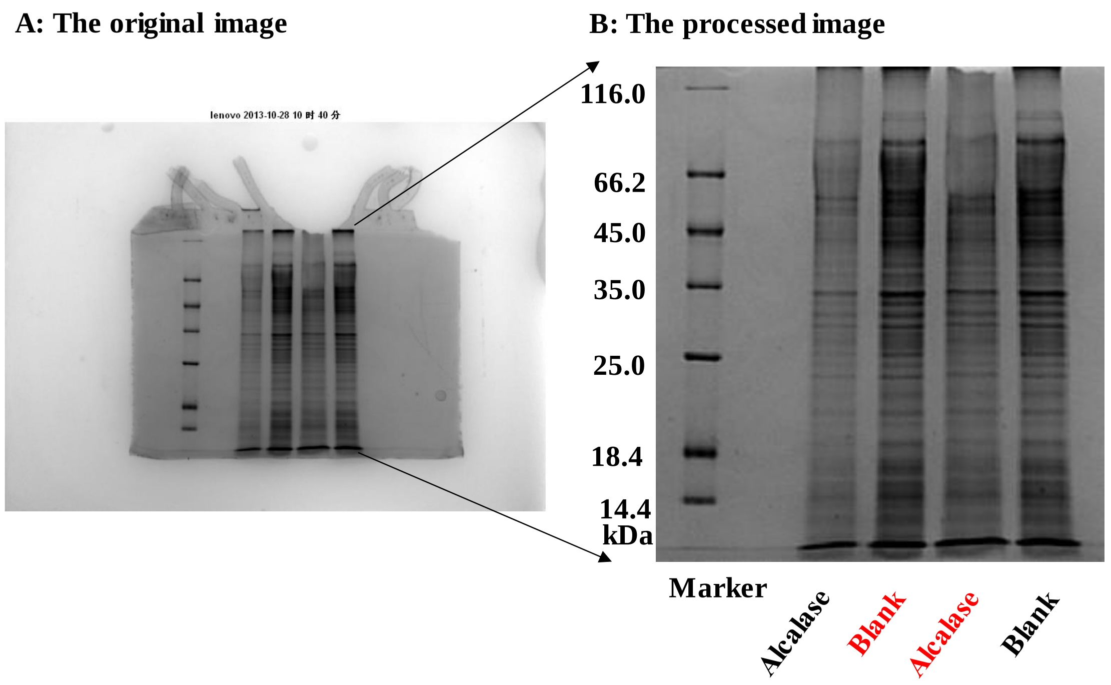


Figure S1. The SDS-page of proteins in the control and Alcalase treated biofilms. A, the original image; B, the processed image.
